# A Review of Mechanisms Underlying Modulation of Cardiorespiratory Endurance and Psychological Stress in College Students by High-Intensity Interval Training (HIIT) Combined with B-Vitamin Supplementation

**DOI:** 10.3390/healthcare13222873

**Published:** 2025-11-12

**Authors:** Jia Fu, Hankai Zhang, Xi Yu, Tian Zhong, Xiaoyu Tao, Ying Xiao

**Affiliations:** 1 Faculty of Medicine, Macau University of Science and Technology, Taipa, Macao SAR, China; 2 School of Health, Zhuhai College of Science and Technology, Zhuhai 519040, China; 3 School of Management, Guangzhou College of Commerce, Guangzhou 510006, China; 4 Department of Lifestyle Medicine, Hong Kong Public Health Technology Research Center, Kowloon, Hong Kong SAR, China

**Keywords:** high-intensity interval training (HIIT), B-vitamin supplementation, psychological stress

## Abstract

University students commonly experience reduced cardiorespiratory fitness and elevated psychological stress due to sedentary lifestyles and academic pressure. Although high-intensity interval training (HIIT) and B-vitamin supplementation are individually known to enhance physiological and psychological health, their combined effects remain underexplored. This systematically guided narrative review synthesizes current evidence on how HIIT and B-vitamin supplementation may interact to influence aerobic capacity, metabolic regulation, and stress resilience. Existing studies demonstrate that HIIT improves VO_2_max, mitochondrial efficiency, and HPA-axis stability, while B-vitamins facilitate energy metabolism, neurotransmitter synthesis, and oxidative stress control. Together, these mechanisms suggest a potential synergistic interaction that enhances endurance and psychological well-being through coordinated metabolic and neuroendocrine pathways. However, this synergy remains a theoretical model requiring empirical validation through controlled trials. The findings highlight the importance of integrated exercise–nutrition strategies in promoting health and resilience among college populations.

## 1. Introduction

Students in modern university environments suffer a double health problem, with a significant decline in cardiorespiratory fitness and increases in psychological stress [1]. Intense academic pressures lead to chronic sedentary lifestyles and reduced physical activity [2]. Meanwhile, study pressures, professional and career uncertainties, and complex interpersonal relationships all contribute to an aggregate burden of mental stress [3], which negatively affects academic performance and mental health. Epidemiological studies show that a high percentage of the student population suffers compromised cardiorespiratory function and high biomarkers for stress, including elevated cortisol levels and self-reported anxiety; the formulation of targeted interventions is therefore needed to prevent adverse health outcomes [4,5]. Despite the extensive literature on exercise and diet, the combined effect of high-intensity interval training (HIIT) and B-vitamin supplementation has not been fully explored.

**Figure 1:**
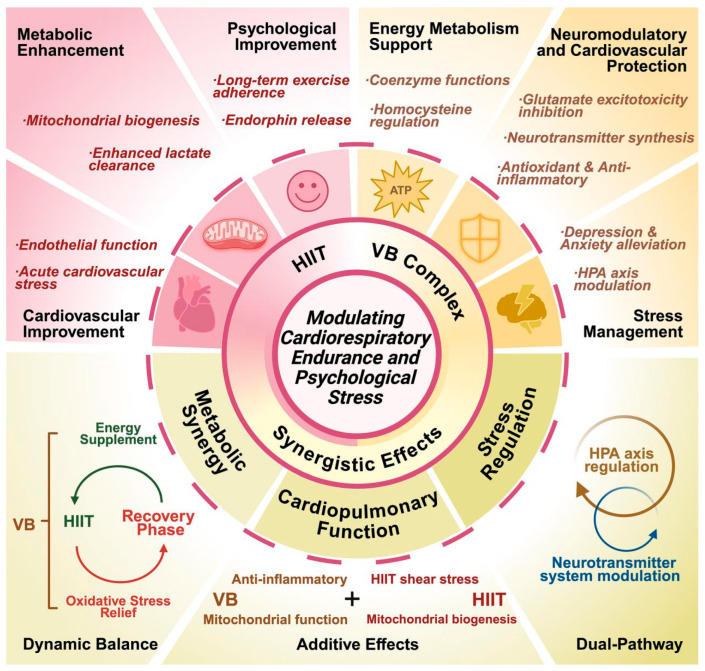
Mechanisms of high-intensity interval training (HIIT) combined with B-vitamin supplementation modulation of cardiorespiratory endurance and psychological stress. Abbreviations: VB: vitamin B, HPA: hypothalamic–pituitary–adrenal.

While a large volume of the published literature has focused either on the physiological effects of individual exercise modalities or the biochemical response to specific nutrients, relatively few studies have addressed potential synergy between the two intervention types [6,7]. This gap in the literature means that an understanding of the potential of such synergy to simultaneously promote physical and psychological health markers is difficult. This oversight risks losing the potential to optimize health, especially with respect to the challenging dual physiological and psychological stressors faced by college students [8,9]. Reducing the twin problems of diminished cardiorespiratory well-being and heightened psychological issues among university students requires novel, interdisciplinary solutions [10,11]. B-vitamins, which play a critical role in energy metabolism and stress response regulation, have the potential to augment the effects of exercise in evoking adaptive physical and mental stress responses [12,13]. Researchers can design health interventions for college students to be more targeted and effective through a better understanding of the biochemical processes underlying this multimodal treatment (Figure 1). Such initiatives foster not just better physical health but also greater academic achievement, ultimately improving quality of life for students and promoting healthier and more balanced lifestyles in university society [14,15].

Accordingly, this review focuses on the interaction between high-intensity interval training (HIIT) and B-vitamin supplementation in improving cardiorespiratory endurance and psychological stress among college students. Although numerous studies have examined either intervention separately, empirical research exploring their combined or synergistic effects remains scarce—particularly in young, healthy populations. Few investigations have concurrently assessed both interventions within the same experimental framework or evaluated how metabolic, cardiovascular, and neuroendocrine mechanisms interact to mediate these outcomes. Addressing this knowledge gap represents the novel contribution of the present review.

Therefore, this paper systematically synthesizes and critically evaluates existing evidence on the independent and combined effects of HIIT and B-vitamin supplementation. It further develops a mechanistic framework describing potential synergistic pathways that integrate metabolic and psychological adaptations. Guided by this objective, the main research question is:

How do HIIT and B-vitamin supplementation—individually and in combination—affect cardiorespiratory endurance and psychological stress in college students, and do these effects demonstrate additive or synergistic interactions?

Finally, the paper is structured as follows: Section 2 outlines the literature search and methodological approach; Section 3 examines the independent physiological and psychological effects of HIIT and B-vitamin supplementation; Section 4 integrates these findings to explain potential synergistic mechanisms; Section 5 discusses research limitations and future directions; and Section 6 presents the conclusions.

## 2. Methods

This review adopts a systematically guided narrative approach grounded in the principles of the Preferred Reporting Items for Systematic Reviews and Meta-Analyses (PRISMA 2020) to ensure methodological transparency and reproducibility.

Rather than performing a meta-analysis, we conducted a structured literature search and qualitative synthesis of studies examining the independent and combined effects of high-intensity interval training (HIIT) and B-vitamin supplementation on cardiorespiratory endurance and psychological stress among college-aged populations.

### 2.1. Literature Search Strategy

A comprehensive search was performed across PubMed, Web of Science, Scopus, and Google Scholar to identify studies published between January 2010 and February 2025.

The search strategy used combinations of Medical Subject Headings (MeSH) and free-text keywords connected by Boolean operators: (“high-intensity interval training” OR “HIIT” OR “interval exercise”) AND (“vitamin B” OR “B-vitamin” OR “thiamine” OR “riboflavin” OR “niacin” OR “pyridoxine” OR “folate” OR “cobalamin”) AND (“college students” OR “young adults”) AND (“cardiorespiratory endurance” OR “VO_2_max” OR “psychological stress” OR “mental health”).

Reference lists of eligible papers and relevant review articles were also manually screened to capture additional studies.

### 2.2. Inclusion and Exclusion Criteria

Studies were included if they met the following criteria:

- Original peer-reviewed empirical research or systematic/narrative reviews addressing either HIIT, B-vitamin supplementation, or both;
- Participants aged 18–30 years or studies involving comparable young adult populations;
- Reported outcomes related to cardiorespiratory endurance, psychological stress, or both;
- Published in English.

Studies were excluded if they:

e. Involved non-human or pediatric samples;
f. Focused exclusively on clinical patients with chronic disease;
g. Were conference abstracts, dissertations, or commentaries lacking empirical data; or
h. Contained insufficient methodological detail for evaluation.

### 2.3. Study Selection Process

The selection process followed the logic of the PRISMA framework. All retrieved records were screened for duplicates, and titles and abstracts were evaluated for relevance. Full texts were reviewed independently by two authors, and disagreements were resolved through discussion until consensus was achieved. For narrative reviews, the study selection process is described textually rather than presented in a full PRISMA diagram, as this review does not include quantitative synthesis.

### 2.4. Data Extraction and Synthesis

Key information was extracted from each included study, including:

- author and publication year;
- participant characteristics;
- intervention type, duration, and dosage;
- outcome measures;
- main findings.

Given the heterogeneity of study designs and outcomes, findings were integrated through qualitative synthesis rather than quantitative pooling.

### 2.5. Risk of Bias and Quality Assessment

Methodological quality and risk of bias were assessed using the AXIS tool (Appraisal Tool for Cross-Sectional Studies), which evaluates study design, sampling, measurement validity, and reporting transparency.

Each study was rated as low, moderate, or high quality.

The overall quality assessment informed the weighting of evidence discussed in later sections.

### 2.6. Quality Assessment

Although this review was not formally registered in an external database such as PROSPERO, all stages of the literature search, study selection, and quality appraisal were documented in detail to ensure transparency and reproducibility. The review process followed PRISMA principles for structured reporting and incorporated explicit quality assessment criteria (see Section 2.5). This approach provides methodological clarity consistent with current standards for narrative evidence syntheses.

## 3. Independent Effects of HIIT and B-Vitamin Supplementation

### 3.1. Independent Effects of HIIT on Cardiorespiratory Endurance and Stress Regulation

High-intensity interval training (HIIT) is a time-efficient training modality characterized by alternating short bursts of vigorous exercise with recovery intervals. It has been extensively documented to induce substantial improvements in maximal oxygen uptake (VO_2_max), a key indicator of cardiovascular fitness and endurance [16,17]. By challenging both aerobic and anaerobic systems, HIIT provokes adaptations that enhance myocardial contractility, vascular responsiveness, and respiratory capacity [18].

HIIT promotes long-term cardiopulmonary adaptations through increased mitochondrial density and oxidative capacity within skeletal muscle cells [19,20]. Such adaptations enable more efficient oxygen utilization and endurance performance. Furthermore, HIIT enhances endothelial function, an essential component of vascular health, by stimulating endothelium-dependent vasodilation and improving blood flow [21,22]. These benefits collectively reduce cardiovascular risk and improve metabolic flexibility.

In addition to its physiological benefits, HIIT exerts important psychological and neuroendocrine effects. It has been associated with reductions in anxiety, perceived stress, and improvements in mood stability [23,24]. HIIT stimulates endorphin and serotonin release, enhances heart rate variability (HRV), and modulates the hypothalamic–pituitary–adrenal (HPA) axis, all of which contribute to stress resilience [25,26].

These findings collectively support HIIT as an efficient modality for improving both cardiorespiratory performance and mental well-being among university populations.

### 3.2. Independent Effects of B-Vitamin Supplementation

B vitamins—including B1 (thiamine), B2 (riboflavin), B3 (niacin), B5 (pantothenic acid), B6 (pyridoxine), B7 (biotin), B9 (folate), and B12 (cobalamin)—serve as essential cofactors in a range of metabolic processes that support cardiovascular health, neurochemical balance, and stress regulation [11,27].

From a cardiovascular perspective, B vitamins contribute to the regulation of homocysteine metabolism, with elevated plasma homocysteine recognized as a major risk factor for vascular dysfunction and atherosclerosis [28,29]. Supplementation with folate, B6, and B12 effectively lowers homocysteine levels, improving endothelial integrity and reducing inflammatory activity, thus preventing cardiovascular damage [28].

In terms of psychological and neuroendocrine regulation, B vitamins play vital roles in neurotransmitter synthesis—notably serotonin, dopamine, and gamma-aminobutyric acid (GABA)—which are central to mood and stress responses [30,31]. Deficiencies in these vitamins are linked to heightened anxiety and depressive symptoms. Clinical trials indicate that supplementation with B6, B9, and B12 improves mood stability, reduces stress-induced emotional fluctuation, and mitigates premenstrual mood disorders [32,33].

Moreover, the antioxidant and anti-inflammatory properties of B vitamins contribute to stress resilience by attenuating oxidative stress and maintaining neuronal integrity [34,35]. Their involvement in methylation reactions also supports DNA repair and gene expression patterns critical for cognitive function and mental health [36,37].

Together, these actions make B-vitamin supplementation a promising non-pharmacological approach to supporting cardiovascular function, mitigating stress, and enhancing mental health among young adults.

### 3.3. Integrative Summary and Transition to Synergy

Collectively, the independent mechanisms of HIIT and B-vitamin supplementation demonstrate distinct yet complementary effects on cardiopulmonary and psychological health. HIIT enhances cardiovascular and metabolic efficiency, while B vitamins support energy metabolism, redox balance, and neurochemical regulation.

These findings suggest a strong biological rationale for interaction between the two interventions. The next section integrates these insights to construct a unified theoretical framework describing how HIIT and B vitamins may act synergistically to improve endurance and stress resilience.

## 4. Synergistic Effects and Mechanistic Evidence of Combined Interventions

Although theoretical models suggest potential synergy between HIIT and B-vitamin supplementation, empirical human evidence directly comparing the combined intervention with each component alone remains limited. Therefore, the following discussion integrates mechanistic insights and indirect findings rather than definitive clinical proof.

### 4.1. Metabolic Support for Synergistic Adaptation

High-intensity interval training (HIIT) imposes substantial metabolic and oxidative demands on skeletal muscle and the cardiovascular system. Adequate B-vitamin availability is essential to sustain these acute challenges. Acting as indispensable cofactors in glycolytic and tricarboxylic acid (TCA) cycle reactions, B vitamins enable efficient adenosine-triphosphate (ATP) generation during repeated high-intensity bouts [38,39]. Consequently, the extent to which HIIT-induced energy turnover translates into improved endurance and stress resilience depends largely on the sufficiency of these coenzymes [15,40].

Mechanistically, vitamins B1 (thiamine), B2 (riboflavin), and B3 (niacin) regulate mitochondrial oxidative phosphorylation through NAD^+^-dependent redox reactions, sustaining cellular energy homeostasis during and after exercise [38,39]. The exercise-driven activation of the AMP-activated protein kinase (AMPK)–PGC-1α axis promotes mitochondrial biogenesis, while B-vitamin-mediated coenzyme availability ensures that these new mitochondria function optimally [15]. By maintaining an appropriate redox state and facilitating electron transport, B vitamins reduce oxidative stress and delay fatigue, providing the biochemical basis for synergistic metabolic adaptation between training and nutrition [40].

Beyond energy production, B vitamins contribute critically to post-exercise recovery. Vitamins B2 and B6 maintain antioxidant enzyme activity and glutathione recycling, thereby limiting oxidative damage from reactive oxygen species (ROS) generated during HIIT [35]. Folate (B9) and vitamin B12 support muscle repair and DNA synthesis following strenuous exercise [13,41], expediting tissue regeneration and minimizing chronic fatigue. This coordinated metabolic support enables faster recovery and sustained high performance.

**Figure 2:**
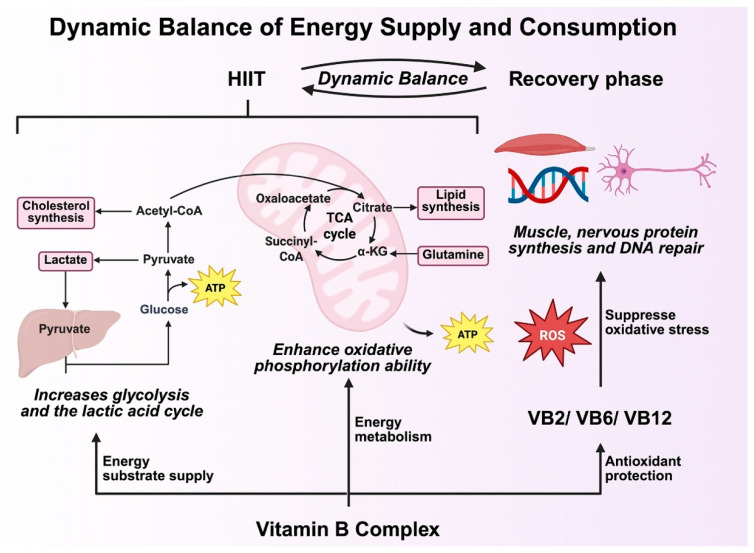
Mechanisms of dynamic balance of energy supply and consumption. Abbreviations: TCA: tricarboxylic acid, CoA: tricarboxylic acid, ROS: reactive oxygen species.

In summary, B-vitamin supplementation does not merely replenish cofactors consumed during exercise; it enhances the adaptive efficiency of HIIT by optimizing energy metabolism, bolstering antioxidant defense, and accelerating recovery. This metabolic foundation underlies the additive and synergistic improvements in cardiopulmonary function and psychological resilience discussed in the following sections (Figure 2).

### 4.2. Additive Effects of Enhanced Cardiopulmonary Function

The combined application of HIIT and B-vitamin supplementation produces additive effects that reinforce aerobic capacity and overall cardiovascular health. HIIT elicits physiological adaptations through repeated cycles of intense exercise and recovery, triggering mitochondrial biogenesis and oxidative phosphorylation that improve skeletal-muscle energy efficiency [42]. This process is primarily mediated through activation of AMP-activated protein kinase (AMPK) and peroxisome-proliferator-activated receptor-gamma coactivator 1-alpha (PGC-1α), key regulators of mitochondrial growth and oxidative metabolism [40]. Consequently, HIIT optimizes oxygen utilization and enhances maximal aerobic power (VO_2_max), a cornerstone of cardiopulmonary endurance.

From a nutritional perspective, B-vitamin supplementation supports these exercise-induced adaptations by maintaining sufficient coenzymatic pools required for oxidative energy production. Vitamin B3 (niacin) serves as a precursor of NAD^+^, an essential redox coenzyme in mitochondrial respiration, while B2 (riboflavin) and B6 (pyridoxine) facilitate electron-transport reactions critical for ATP generation [41]. Elevated NAD^+^ levels have been shown to enhance mitochondrial function and reduce metabolic fatigue, thereby complementing the endurance improvements obtained from HIIT [13]. This integration of metabolic and exercise stimuli maximizes the efficiency of cardiopulmonary function and promotes long-term aerobic conditioning.

The additive benefits extend to vascular and endothelial health. HIIT induces shear-stress-mediated endothelial nitric-oxide-synthase (eNOS) activation, which improves vasodilation and microcirculatory function [21,22]. When combined with B-vitamin supplementation, the overall improvement in vascular reactivity surpasses that achieved by either intervention alone. B-vitamins, particularly folate (B9) and B12, lower plasma homocysteine—a key pro-inflammatory metabolite—thereby reducing oxidative damage and maintaining endothelial integrity [28]. This dual mechanism—exercise-induced mechanical adaptation and nutrient-driven biochemical protection—creates an additive shield against cardiovascular dysfunction and chronic inflammation.

In addition, the HIIT and B-vitamin combination attenuates oxidative stress, another determinant of vascular and cardiopulmonary health. Although acute HIIT temporarily elevates reactive oxygen species (ROS), regular training strengthens endogenous antioxidant defenses, while B-vitamins support glutathione metabolism and ROS scavenging [35]. Such coordination ensures that oxidative balance is restored efficiently after exercise, accelerating recovery and maintaining vascular elasticity. Together, these adaptations translate into enhanced cardiac efficiency, improved oxygen delivery, and greater resilience against fatigue and oxidative injury.

**Figure 3:**
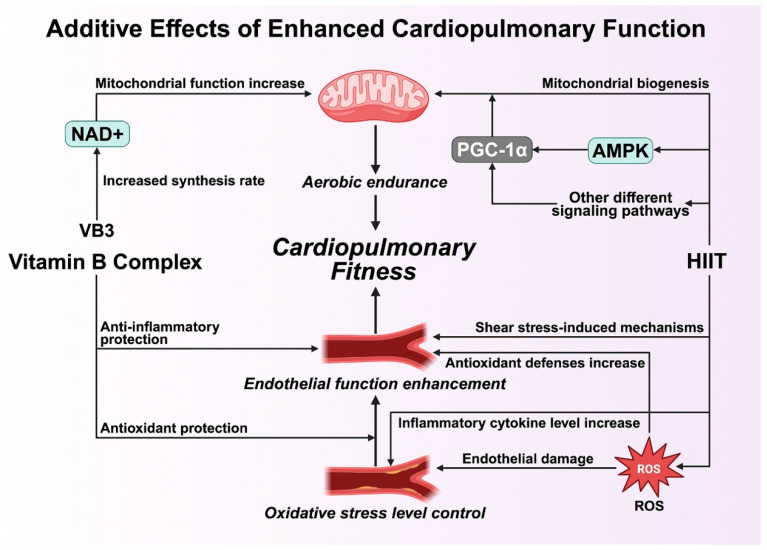
Mechanisms of additive effects of enhanced cardiopulmonary function. Abbreviations: AMPK: AMP-activated protein kinase; NAD^+^: nicotinamide adenine dinucleotide.

Finally, the additive interaction between HIIT and B-vitamin supplementation establishes a multidimensional framework for cardiovascular optimization. By synchronizing exercise-induced mitochondrial biogenesis, redox regulation, and endothelial stabilization with nutrient-mediated metabolic support, the combined intervention provides comprehensive enhancement of cardiopulmonary function. This synergy underscores the potential for integrated exercise–nutrition strategies to achieve superior aerobic and vascular outcomes compared with either modality alone (Figure 3).

### 4.3. Dual-Pathway Mechanism for Stress Regulation

Beyond physiological outcomes, the synergistic interaction between HIIT and B-vitamin supplementation also influences neuroendocrine and psychological regulation. HIIT modulates the HPA axis, balancing cortisol secretion and promoting neuroplastic adaptation to repeated stressors [25,43]. These adaptations reduce anxiety-like responses and improve emotional control. Concurrently, B vitamins, particularly B6, B9, and B12, facilitate neurotransmitter synthesis (serotonin, dopamine, and GABA), contributing to improved cognitive stability and mood regulation [30,44].

Combined, these mechanisms exert dual-pathway stress modulation: the physiological pathway through HPA-axis regulation and the biochemical pathway through neurotransmitter synthesis. This synergy enhances psychological resilience and reduces perceived stress levels [45,46].

**Figure 4:**
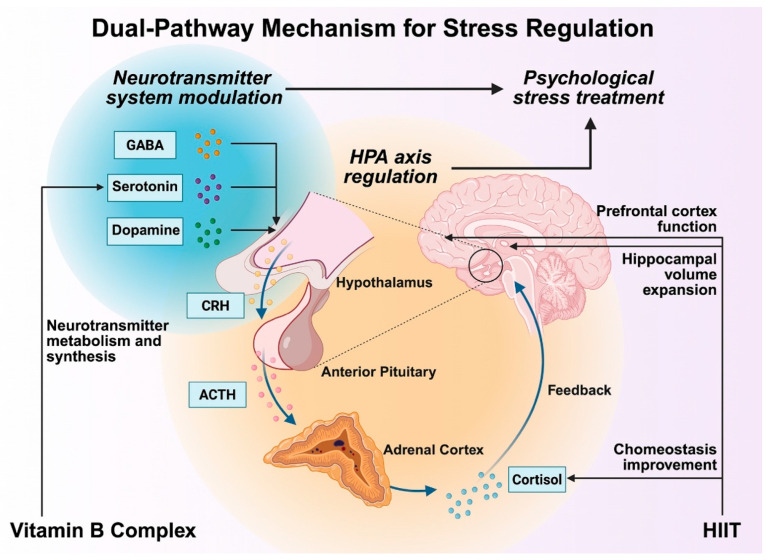
Outline of a dual-pathway mechanism for stress regulation. Abbreviations: GABA: gamma-aminobutyric acid; CRH: corticotropin-releasing hormone; ACTH: adrenocorticotropic hormone.

In college populations, such combined adaptations may buffer academic and lifestyle stress, offering an effective non-pharmacological strategy for improving both mental and physical well-being (Figure 4).

### 4.4. Limitations of Current Evidence

Despite the conceptual rationale for combining high-intensity interval training (HIIT) and B-vitamin supplementation, empirical evidence on their concurrent effects remains limited. Most existing data are derived from separate investigations of exercise physiology and nutritional biochemistry, and no randomized controlled trials have directly compared the combined intervention with single-modality controls in college populations. As a result, the conclusions regarding potential synergy are largely inferential, drawn from converging mechanisms such as mitochondrial biogenesis, HPA-axis modulation, and oxidative-stress regulation.

While several cited studies were conducted in older or clinical populations, they nonetheless provide valuable mechanistic insights into HIIT–B-vitamin interactions. However, extrapolating these findings to healthy young adults must be approached with caution due to differences in metabolic, cardiovascular, and psychological baselines. Future research should therefore prioritize controlled clinical trials to determine whether these mechanistic overlaps translate into measurable additive or synergistic effects in practice.

It should also be emphasized that no direct meta-analytic or empirical validation currently supports the proposed interaction model. The mechanisms summarized in Figure 1, Figure 2, Figure 3 and Figure 4 represent a conceptual synthesis derived from existing physiological and biochemical literature rather than experimentally verified outcomes. Accordingly, these models should be interpreted as theoretical hypotheses that require verification through prospective, longitudinal, and multi-factorial studies.

Finally, this review contributes an integrative perspective by consolidating evidence from diverse methodological approaches and applying explicit quality assessment criteria (see Section 2.6), thereby enhancing the transparency and reproducibility of the evidence synthesis while acknowledging its inherent limitations.

## 5. Future Research Directions

Although the current evidence provides a theoretical framework for the synergistic benefits of HIIT and B-vitamin supplementation, substantial research gaps remain. Addressing these limitations requires methodological refinement, integration of mechanistic biomarkers, and population-specific validation. Future studies should prioritize designs that empirically evaluate the combined intervention rather than extrapolating from the independent effects of exercise and nutrition.

### 5.1. Methodological Improvements and Study Design

Future experimental designs should include parallel-group RCTs comparing HIIT, B-vitamin supplementation, and their combination, with standardized intervention duration (8–12 weeks) and mechanistic endpoints. Existing studies have primarily examined HIIT and B-vitamin supplementation in isolation. To establish a robust evidence base for their synergy, randomized controlled trials (RCTs) directly comparing (1) HIIT only, (2) B-vitamin supplementation only, and (3) combined HIIT + B-vitamin interventions are needed [11,16]. Such trials should employ well-defined protocols regarding training frequency, intensity, and duration, along with standardized vitamin dosages and supplementation periods [31,32].

Moreover, sample homogeneity should be ensured to avoid confounding by age, baseline fitness, and dietary patterns. Stratified randomization based on sex, body mass index (BMI), and habitual activity levels can reduce variability. Future trials should also incorporate follow-up assessments to evaluate the persistence of synergistic benefits beyond the training period, particularly concerning long-term adaptation and stress resilience.

A critical methodological improvement involves the inclusion of multi-modal outcome measures. Traditional physiological endpoints such as VO_2_max, heart rate variability (HRV), and endothelial function should be accompanied by biochemical markers (e.g., cortisol, catecholamines, oxidative stress indices, and inflammatory cytokines) and psychological scales (e.g., the Perceived Stress Scale, Beck Anxiety Inventory, or Profile of Mood States). Such integrated evaluation would allow the quantification of physical–psychological synergy in response to combined interventions [34,35].

### 5.2. Mechanistic and Translational Research Priorities

Potential molecular targets such as AMPK–PGC-1α signaling, NAD^+^/NADH redox balance, and HPA-axis regulation should be investigated using omics-based and biochemical analyses. Future research should move beyond descriptive outcomes to probe molecular and cellular mechanisms underlying the HIIT–B-vitamin interaction. Experimental studies may employ metabolomic and transcriptomic profiling to examine how vitamin-dependent pathways (e.g., NAD^+^/NADH redox balance, AMPK–PGC-1α signaling, and methylation cycles) are modulated by exercise [38,39].

Animal models or controlled human trials could further investigate neuroendocrine responses, such as HPA-axis reactivity and neurotransmitter synthesis regulated by vitamin B6 and B12 [30,36].

Translational research should also examine the optimal dosage and combination ratios of B vitamins for supporting HIIT-induced adaptations. While current supplementation trials often use generalized multivitamin formulations, precision-nutrition studies could explore individualized dosing based on metabolic needs, genetic polymorphisms (e.g., MTHFR), and lifestyle factors [37].

Additionally, integrating wearable technologies and digital biomarkers (heart rate, oxygen saturation, perceived exertion) can provide real-time feedback for adaptive intervention design.

### 5.3. Population-Specific and Contextual Considerations

College students represent a unique population characterized by psychological stress, irregular routines, and suboptimal nutrient intake; future interventions should therefore tailor B-vitamin dosage and HIIT intensity to these contextual factors. Most of the current data are derived from general adult or athletic populations. However, college students—the target population of this review—represent a unique group with distinct physiological and psychological challenges. They experience chronic academic stress, irregular sleep patterns, and fluctuating physical activity levels [23,24].

Future interventions should therefore adopt ecologically valid designs, integrating HIIT sessions into academic schedules and assessing how vitamin supplementation influences compliance, recovery, and academic performance.

Cross-cultural studies are also encouraged, as nutritional habits, stress perception, and exercise preferences differ widely across regions. Comparative trials in Asian, European, and American college populations could reveal whether synergistic effects are moderated by lifestyle, diet, or environmental factors.

Furthermore, attention should be given to gender-specific responses, as hormonal differences may influence both metabolic adaptation and psychological outcomes.

### 5.4. Conceptual and Ethical Framework

To ensure reproducibility and transparency, future reviews should follow PRISMA and Cochrane guidelines, register protocols in public databases such as PROSPERO, and conduct quality assessments using PICO or AXIS frameworks [19,28].

Researchers must also consider ethical dimensions, including informed consent, mental health monitoring during HIIT programs, and ensuring safe vitamin dosages in young adults.

## 6. Conclusions

This study presents a systematic narrative review that integrates existing empirical findings to evaluate how the combined and independent effects of high-intensity interval training (HIIT) and B-vitamin supplementation influence two interrelated aspects of student health: cardiorespiratory endurance and psychological stress regulation.

Rather than reporting new experimental data, this review synthesizes evidence from exercise physiology and nutritional neuroscience to provide mechanistic insights into how metabolic and neuroendocrine pathways may underpin their potential synergy.

The synergistic health effects of HIIT and B-vitamin supplementation constitute a useful intervention program for enhancing cardiopulmonary resilience and psychological strength among university students. In light of increasingly sedentary lives and increasingly high levels of stress among this population, a twin approach of physical and psychological wellness is useful. HIIT has proven benefits in significantly augmenting aerobic capacity, mitochondrial function, and cardiovascular performance, and is therefore an ideal method of improving endurance and fitness. With its capacity for balancing autonomic nervous system function and HPA axis modulation, HIIT represents a vital resource in curtailing psychological strain and maximizing resilience. Together with all such adaptations, HIIT qualifies as an ideal strategy for students who have little time to exercise.

B vitamins—as cofactors in energy metabolism and essential processes such as neurotransmitter production and oxidative stress management—complement HIIT effects by optimizing metabolic processes, helping avoid exhaustion and reversing oxidative damage due to exercise. Their function in homocysteine metabolism, mitochondrial metabolism, and neuroprotection makes them beneficial in cardiovascular and neurological health. Their ability to modulate cognition and mood stability suggests another benefit in stress management.

The synergistic action of HIIT and B-vitamin supplementation entails interacting mechanisms of action, including the upregulation of energy metabolism, repression of oxidative stress, improvement of endothelial function, and neuroendocrine regulation. This synergistic approach is an effective, evidence-based intervention for students and populations with similar demographics.

While promising, further research is needed to define the optimal training parameters, dosing regimens, and personalized strategies for maximizing the effectiveness of this dual therapy. Future research should aim to understand the genetic and metabolic variability influencing the interindividual responses to HIIT and B-vitamin supplementation, leveraging advances in novel precision nutrition and health monitoring technologies coupled with AI.

While both high-intensity interval training (HIIT) and B-vitamin supplementation independently enhance cardiometabolic and psychological health, their combined synergistic potential remains theoretical, supported mainly by mechanistic reasoning rather than direct empirical data. Future randomized controlled trials and meta-analytic studies are required to validate these interactions under controlled conditions and to quantify their additive benefits. Taken together, HIIT and B-vitamin supplementation represent complementary, evidence-informed strategies for promoting physical endurance and psychological resilience in young adults.

## Data Availability

No new data were created or analyzed in this study. Data sharing is not applicable to this article.
